# Late gadolinium uptake demonstrated with magnetic resonance in patients where automated PERFIT analysis of myocardial SPECT suggests irreversible perfusion defect

**DOI:** 10.1186/1471-2342-8-17

**Published:** 2008-12-12

**Authors:** Lene Rosendahl, Peter Blomstrand, Jan L Ohlsson, Per-Gunnar Björklund, Britt-Marie Ahlander, Sven-Åke Starck, Jan E Engvall

**Affiliations:** 1 Dept. of Clinical Physiology, County Hospital Ryhov, SE-55185 Jönköping, Sweden; 2 Center for Medical Image Science and Visualization, CMIV, Linköping University Hospital, SE-58185 Linköping, Sweden; 3 Radiology, County Hospital Ryhov, SE-55185 Jönköping, Sweden; 4 Hospital Physics Unit, Dept. Of Oncology, County Hospital Ryhov, SE-55185 Jönköping, Sweden; 5 Dept. of Clinical Physiology, Linköping University Hospital, SE-58185 Linköping, Sweden

## Abstract

**Background:**

Myocardial perfusion single photon emission computed tomography (MPS) is frequently used as the reference method for the determination of myocardial infarct size. PERFIT^® ^is a software utilizing a three-dimensional gender specific, averaged heart model for the automatic evaluation of myocardial perfusion. The purpose of this study was to compare the perfusion defect size on MPS, assessed with PERFIT, with the hyperenhanced volume assessed by late gadolinium enhancement magnetic resonance imaging (LGE) and to relate their effect on the wall motion score index (WMSI) assessed with cine magnetic resonance imaging (cine-MRI) and echocardiography (echo).

**Methods:**

LGE was performed in 40 patients where clinical MPS showed an irreversible uptake reduction suggesting a myocardial scar. Infarct volume, extent and major coronary supply were compared between MPS and LGE as well as the relationship between infarct size from both methods and WMSI.

**Results:**

MPS showed a slightly larger infarct volume than LGE (MPS 29.6 ± 23.2 ml, LGE 22.1 ± 16.9 ml, p = 0.01), while no significant difference was found in infarct extent (MPS 11.7 ± 9.4%, LGE 13.0 ± 9.6%). The correlation coefficients between methods in respect to infarct size and infarct extent were 0.71 and 0.63 respectively. WMSI determined with cine-MRI correlated moderately with infarct volume and infarct extent (cine-MRI vs MPS volume r = 0.71, extent r = 0.71, cine-MRI vs LGE volume r = 0.62, extent r = 0.60). Similar results were achieved when wall motion was determined with echo. Both MPS and LGE showed the same major coronary supply to the infarct area in a majority of patients, Kappa = 0.84.

**Conclusion:**

MPS and LGE agree moderately in the determination of infarct size in both absolute and relative terms, although infarct volume is slightly larger with MPS. The correlation between WMSI and infarct size is moderate.

## Background

Coronary artery disease (CAD) is currently the leading cause of death in industrialized countries and a growing concern in low- and middle-income countries [1]. Infarct size is a powerful prognostic predictor for survival in patients with CAD and left ventricular (LV) dysfunction [2,3]. It forecasts the risk of developing heart failure, which in turn influences the choice of medical treatment, catheter intervention or surgery. Hypokinetic left ventricular wall segments with stunned or hibernating myocardium are viable and function may return after revascularization. The potential reversibility of chronic LV dysfunction is thus an important consideration in patients with heart failure [4]. Moreover, infarct size may be an attractive surrogate end point in studies of infarct limiting interventions after acute myocardial infarction [5-7].

Myocardial perfusion single photon emission computed tomography (MPS) has often been used as a reference method to estimate infarct size [8], based on visual qualitative evaluation of the perfusion defect. Objective measurement and standardized evaluation are desirable in the application of all cardiac imaging methods. Hoffmann et al [9] showed that physicians employed in the same echo lab agreed well in wall motion assessment but less so when compared with physicians trained in other hospitals. Computer-assisted assessment of infarct size in MPS imaging may reduce the variability between different observers [10,11]. PERFIT^® ^(HERMES Medical Solutions, Stockholm, Sweden) is an automatic software package for quantitative analysis of infarct size and severity. The reference is a three-dimensional, gender specific, averaged heart, generated from a defined reference population [12,13].

Late gadolinium enhancement magnetic resonance imaging (LGE) accurately determines infarct size [14] and has a high reproducibility [15]. A high spatial resolution enables measuring infarct transmurality and from this parameter assessment of viable myocardium is possible [16-18].

Previous studies have shown a good agreement between infarct size assessed with MPS and LGE [6,19-21]. The purpose of the present study was to compare the perfusion defect size by MPS, assessed with an automated infarction size evaluation software, PERFIT, with the hyperenhanced volume measured with LGE, in patients with a perfusion defect suggestive of infarction on MPS. The relationship between ventricular function and infarct size as well as the major coronary supply to the infarct area were also compared between the methods.

## Methods

### Study Population

Forty patients, 33 men and 7 women, average age 65 ± 10 years (range 36 – 84) were consecutively enrolled between June 2002 and March 2004. Thirty-two of these patients had been diagnosed with myocardial infarction and the remaining 8 had symptoms suggestive of coronary artery disease. Fourteen patients had been revascularized, either with open heart surgery or percutaneous coronary interventions. Patients referred for MPS with suspicion of coronary artery disease were included in the study if they had an irreversible uptake reduction suggesting a myocardial scar. Exclusion criteria were contraindications for magnetic resonance imaging (MRI) such as an implantable cardiac device, ferromagnetic clips, claustrophobia, or an intercurrent cardiovascular event between the studies such as revascularization or myocardial infarction. No patient was excluded because of technical failure or poor image quality. MPS and LGE were performed within 42 ± 34 days (range 10 – 192). The study was approved by the Ethics committee at Linköping University and complied with the Declaration of Helsinki. All patients gave informed consent.

### Myocardial perfusion imaging

The rest images from a two-day stress/rest protocol were used. Imaging at rest was performed 2 – 3 days after stress imaging using 8.6 MBq ^99m^Tc tetrofosmin/kg bodyweight (max 860 MBq) (Amersham Health, Buckinghamshire, UK). A dual-detector gamma camera (E. CAM, Siemens Medical Systems Inc, Hoffman Estates, Il, USA) equipped with a high resolution collimator was used. Thirty-two views were acquired in steps of 2.8 degrees per detector and the acquisition time/angle was 25 s. (In the first nine patients, 16 views per detector were used with an acquisition time/angle of 50s). A 19% window was "asymmetrically placed" (129 – 155 keV) on the 140 keV peak. A 64*64 word matrix with a pixel size of 6.6 mm was used. The acquisition files were reconstructed by the nuclear technicians using filtered back-projection prefiltered with a Butterworth filter (cut off 0.8/cm, order 10) (Hermes Medical Solutions, Stockholm, Sweden) and the short-axis slices were manually re-oriented perpendicular to the cardiac long axis. In case of interfering bowel uptake, acquisition was repeated after intake of fluids. The result of the reconstruction procedure was controlled and the images were initially analyzed and reported for clinical patient care by four experienced nuclear physicians, whose individual evaluations were the basis for inclusion in the study. After inclusion, images at rest were reanalyzed with PERFIT by one expert reader without knowledge of the LGE results. PERFIT compares manually reconstructed transversal slices using an automated image alignment algorithm with a three-dimensional gender specific, averaged heart model, generated from a defined reference population. The software assesses the size of the myocardial scar (ml), the infarct extent (% of myocardial volume) and analyzes the percentage of each coronary artery perfusion area involved [12,13]. The threshold for MPS scar is set to an absolute level of < 2 SD of the highest perfusion uptake in a remote area of the myocardium. In the PERFIT analysis, the image alignment by the software was visually checked but no manual correction of the automatic fit was necessary. This totally automatic volumetric MPS technique was chosen in favour of polar-maps since the presentation of the short-axis slices is identical to that of LGE, thus allowing a simplified visual infarct interpretation.

### Magnetic resonance imaging

The patients were placed in the magnet (1.5 T MagnetomVision, Siemens, Erlangen, Germany) in supine position. A circular polarized body-array surface coil was used in all measurements. ECG-triggered MR images were obtained during repeated breath-holds. Cine-MR imaging attempted to cover the entire left ventricle with on average 9 (range 7 – 11) short-axis slices and three long axis planes (2, 3 and 4 chamber views). A turbo-fast low angle shot (FLASH) sequence with repetition time 100 ms and echo time 4.8 ms was used. The contrast-enhanced images were acquired at the same slice positions as the cine-images. Gadopentetate dimeglumine (Gd-DTPA) 0.2 mmol/kg bodyweight (Schering Nordiska AB, Järfälla, Sweden) was administered in 33 patients and 0.1 mmol/kg bodyweight in the initial seven patients. A segmented IR turbo-FLASH-sequence was used, with a repetition time determined by 2 R-R intervals, an echo time of 3.4 ms and an inversion time of 175–250 ms with 300 ms delay after the R-wave. Slice thickness was 8 mm, intersection gap 2 mm, field-of-view 270 × 360 mm and image matrix 132 × 256. The segmented sequence acquired 33 k-space lines following the inversion pulse. A 300 ms delay forced the data acquisition to the diastolic phase. The total acquisition time per slice was 10 heartbeats including one magnetization steady-state preparation period. Optimal contrast between hyperenhanced areas and normal myocardium was established by continually adjusting the inversion time to null the signal from the healthy myocardium. The CNR (contrast-to-noise ratio) obtained was 6.8 ± 3.3.

Left ventricular myocardial volume and infarct size were measured twice on the LGE images by two observers, both without knowledge of the results from the MPS measurements. Segmentation of the myocardium and the infarct area was performed manually with planimetry using ImageJ 1.29× (Wayne Rasband, NIH USA, ). The infarct area was set to a signal intensity exceeding 2 SD. of the signal intensity in a remote area of the myocardium. Infarct extent was calculated as the percentage of scar compared with the total volume of the myocardium. Long axis views aided the determination of scar. The papillary muscles were included in the LV size/infarction size if they were attached to the myocardium at that particular site. The volume of the infarct and of the healthy myocardium was averaged from the two observers and infarct size was expressed as volume and extent. Problems with partial volume effects, due to thick slices, in the apex and in the left ventricular outflow tract were resolved by consensus (6% of all slices). Wall motion scoring was performed in the same manner as with echocardiography (see below).

### Echocardiography

Left ventricular function was determined with echocardiography (Siemens Sequoia 256, Siemens Healthcare Inc, Mountainview, CA, USA) on the same day as the MRI examination. The patients were investigated in the left lateral position, with 2D recordings from the apical and parasternal positions. Cineloops were digitized and stored on magneto-optic discs (MO) as well as 10s views stored on video tape. The left ventricle was divided into 16 segments [22]. Wall motion was determined by two independent observers and the mean value was applied to each segment. Wall motion scoring used the following semi quantitative values: normal = 1, hypokinetic = 2, akinetic = 3, dyskinetic = 4, aneurysm = 5. Scores were summed and divided by the 16 segments to obtain a global wall motion score index for the left ventricle for each patient.

### Coronary artery supply area

In the 33 patients where an infarct scar was seen on both MPS and LGE, the major coronary artery supply for the infarcted segments in LGE was manually determined according to a 16 myocardial segmental model that closely resembles the presently recommended 17-segment model [22,23]. The left ventricle was divided into equal thirds perpendicular to the long axis of the heart. A mid-slice in each third was selected and the segmental scar area calculated after manually outlining the epi- and endocardial borders [24]. The late gadolinium positive fraction in each coronary artery perfusion area was calculated, provided the percentage involvement of any segment exceeded 5%. All segments were assumed to be of equal size. The coronary perfusion area with the highest infarct fraction was assumed to be perfused by the infarct related artery. For MPS, the software gave a figure of infarcted volume for each coronary artery perfusion area. The coronary perfusion area with the highest infarct fraction was assumed to be the infarct related artery.

### Statistical analysis

Analyses were performed using SPSS 13.0 (SPSS Inc, Chicago, Illinois). Values are reported as mean ± SD. For infarct size and extent, two-sided t-test for paired observations was used. Correlation coefficients and related p values are reported and Bland-Altman plots used. Kappa statistics was used to determine the correspondence between the two methods in the determination of the major vascular supply of the infarcted region.

## Results

**Figure 1:**
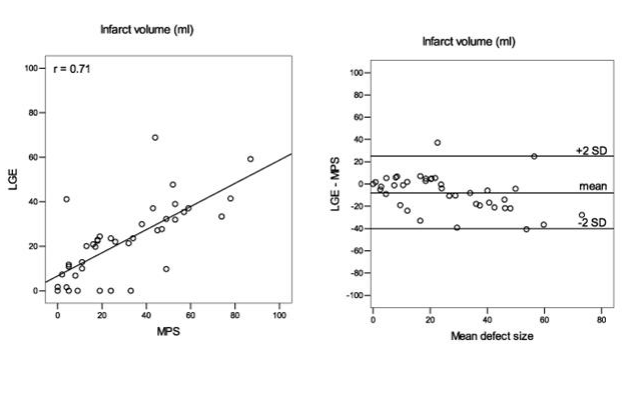
**Infarct volume**. Determination of infarct volume. LGE vs MPS (left). Regression line is shown. Corresponding Bland-Altman plot (right). Values are averaged from two observers.

**Figure 2:**
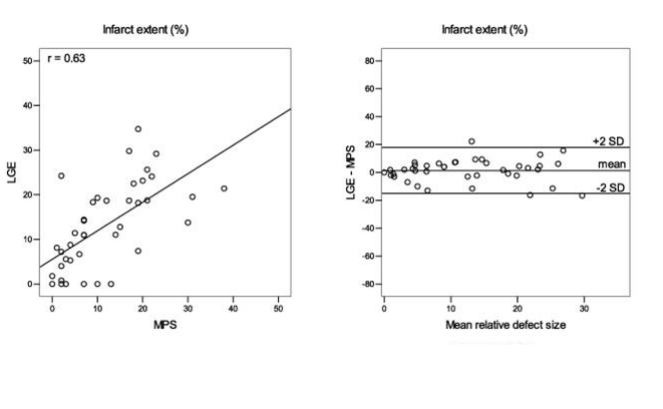
**Infarct extent**. Determination of infarct extent. LGE vs MPS (left). Regression line is shown. Corresponding Bland-Altman plot (right). Values are averaged from two observers.

The myocardial infarct volume assessed with MPS was 29.6 ± 23.2 ml (range 0 – 87) compared with LGE 22.1 ± 16.9 ml (range 0 – 69), p = 0.01, with a correlation coefficient of *r *= 0.71, figure 1 and table 1. Infarct extent was 11.7 ± 9.4% (range 0 – 38) using MPS and 13.0 ± 9.6% (range 0 – 35) with LGE, p = 0.32, with a correlation coefficient of *r = *0.63, figure 2. The intraobserver variability for LGE infarct volume was 0.3 ± 8.0 ml and 0.2 ± 6.4 ml respectively for the two observers and for LGE infarct extent 0.1 ± 4.3% and 0.2 ± 3.8 respectively. Interobserver variability for LGE infarct volume was 1.0 ± 3.0 ml (p = 0.05) and for infarct extent 0.3 ± 2.4% (p = 0.4).

**Table 1:** Infarct volume and infarct extent

	**MPS**	**LGE**	**Difference**	**95% CI for difference**	**r**	**p**
**Infarct volume (ml)**	29.6	22.1	-7.5	-12.7 to -2.3	0.71	0.01
**Infarct extent (%)**	11.7	13.0	1.3	-1.3 to 3.9	0.63	0.32

MPS automatic calculation and LGE averaged from manual measurements of 2 observers.

**Figure 3:**
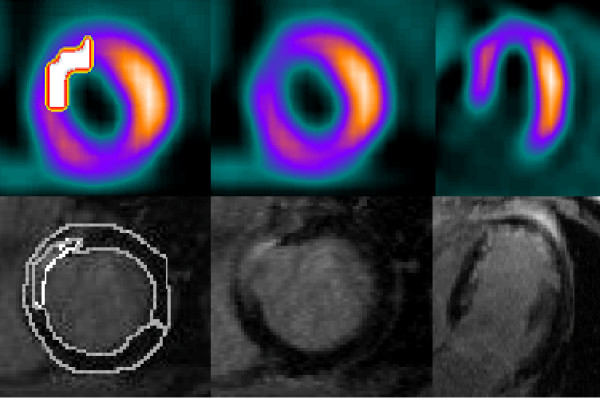
**Subendocardial anteroseptal infarct where MPS and LGE agree**. Upper row: Left – evaluation of the scar by MPS(Perfit) in short-axis view. Middle – corresponding image without automatic scar determination. Right – corresponding long axis view. Lower row: Left – manual evaluation of the scar on LGE image in short axis view. Middle – corresponding image without manual evaluation. Right – corresponding long axis view.

**Figure 4:**
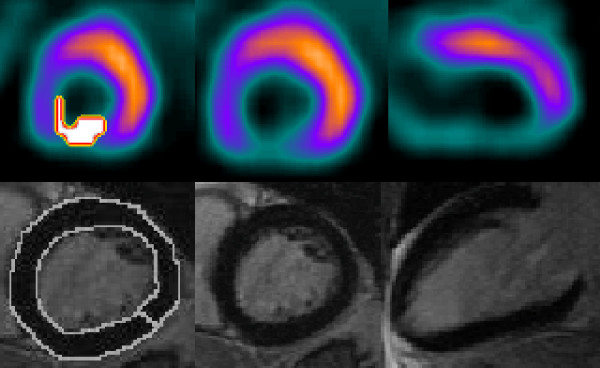
**Discordance between MPS and LGE. MPS shows inferior infarct whereas LGE shows normal myocardium**. For explanation see figure 3.

PERFIT and LGE were concordant in 34 patients, while there were differences in six patients. In five patients with normal LGE results, PERFIT and clinical evaluation reported myocardial perfusion defects. A retrospective review of these MPS studies showed that attenuation defects were possible. In one patient, PERFIT did not detect a scar that was reported from clinical MPS evaluation and with LGE. Additionally, clinical MPS erroneously reported a scar in one patient that was cleared with PERFIT as well as LGE, see table 2, figures 3 and 4. If these 7 patients were removed from the analysis, infarct volume assessed with MPS was 33.2 ± 23.5 ml and with LGE 26.7 ± 14.9 ml, (p = 0.04). Infarct extent was 13.2 ± 9.6% with MPS and 15.7 ± 8.3% with LGE, (p = 0.08). The correlation coefficient between the two methods was in this subset 0.70 for infarct volume and 0.59 for infarct extent.

**Table 2:** Concordance between MPS and LGE

	**LGE normal**	**LGE pathologic**	**Sum**
**MPS(Perfit) normal**	1	1	**2**
**MPS(Perfit) pathologic**	5	33	**38**
**Sum**	**6**	**34**	**40**

Normal and pathologic results reported with the two methods.

Contrary to the general impression, in three patients, the LGE analysis suggested a larger infarct volume and/or extent than MPS (> 1 SD of their difference). In two of these cases, the infracted area included the apex which made delineation of the endocardium difficult due to low contrast between the infarct area and the signal in the blood pool. In the third patient, bowel uptake on MPS-images interfered with the interpretation of the inferior perfusion reduction. In six studies MPS showed a larger infarct volume and/or extent compared with LGE (> 1 SD). In two of these studies MPS showed a reduced uptake where LGE showed thin walls and in four patients MPS showed reduced inferior uptake where LGE showed a small inferior infarct or no scar at all. In three of these four patients both MPS and LGE displayed dilated left ventricles.

**Figure 5:**
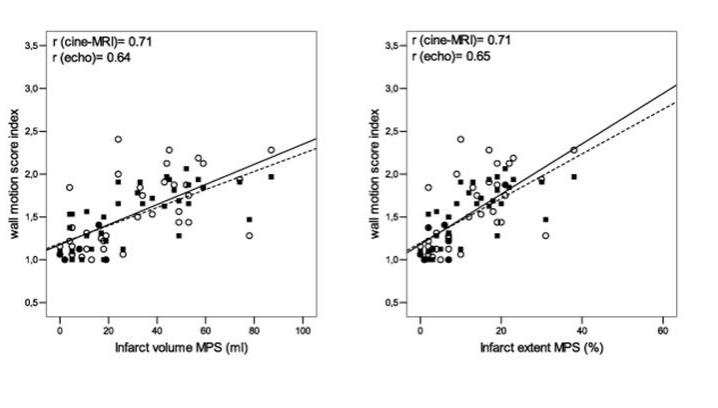
**Wall motion score index vs infarct volume and extent, assessed with MPS**. Wall motion score index determined by cine-MRI and echocardiography vs infarct volume assessed by MPS, in ml (left) and expressed as percent of myocardial volume (right). ▪ = WMSI(cine-MRI), ◦ = WMSI(echo). Dotted line expresses the regression line for WMSI(cine-MRI) and solid line the regression line for WMSI(echo).

**Figure 6:**
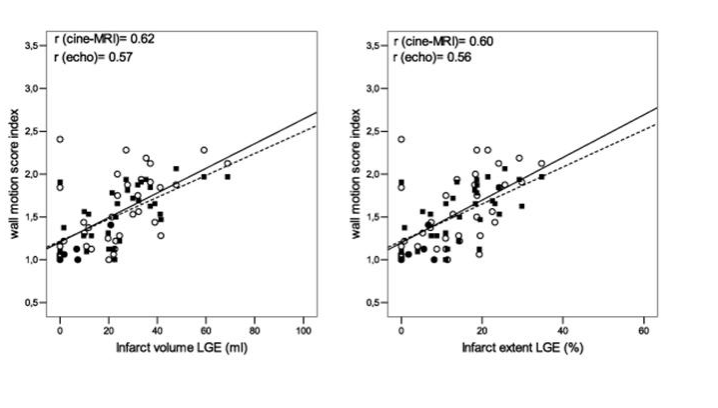
**Wall motion score index vs infarct volume and extent, assessed with LGE**. Wall motion score index determined by cine-MRI and echocardiography vs infarct volume assessed by LGE, in ml (left) and expressed as percent of myocardial volume (right). ▪ = WMSI(cine-MRI), ◦ = WMSI(echo). Dotted line expresses the regression line for WMSI(cine-MRI) and solid line the regression line for WMSI(echo).

Wall motion score index determined with cine-MRI and with echo correlated reasonably well with infarct volume and extent with both MPS and LGE. The correlation WMSI(cine-MRI) versus infarct volume(MPS) was r = 0.71 and infarct extent(MPS) r = 0.71. WMSI(echo) vs infarct volume(MPS) was r = 0.64 and for infarct extent r = 0.65, respectively, figure 5. WMSI (cine-MRI) vs infarct volume(LGE) was r = 0.62 and infarct extent(LGE) r = 0.60. WMSI(echo) versus infarct volume (LGE) was r = 0.57 and infarct extent r = 0.56 respectively, figure 6.

In the 33 scans where MPS and LGE both showed myocardial scars, PERFIT determined that LAD was the main coronary artery supply of the infarcted segments in 21 scans (LAD territory involvement in a total of 25 exams), whereas LCx and RCA were the main coronary artery supply in 6 scans each (LCx and RCA territory involvement in a total of 19 and 16 exams respectively). Corresponding figures with LGE were for LAD 20 scans (LGE uptake in a total of 107 segments) and for LCx and RCA 5 and 8 scans respectively (LGE uptake in a total of 63 segments and 71 segments respectively). Hence, there was a good agreement in 30 of the 33 scans with myocardial damage, (Kappa = 0.84).

## Discussion

In this study, the mean infarct volume from MPS, evaluated with a fully automatic computer software, was slightly larger than manually delineated LGE. This is in line with previous results, usually obtained with visual expert readings of either standard tomographic projections or polar map displays. PERFIT is fast and does not seem to miss clinically relevant hypoperfused areas. The Achilles heel of any automated perfusion software is the issue of breast and abdominal attenuation. In a gated SPECT presentation, the absence of wall motion disturbance suggests attenuation rather than scar. Despite the lack of such checks and balances in PERFIT, the agreement with LGE is in line with conventional methods. Medrano et. al [25] compared the sestamibi perfusion defect with the histological evaluation of infarct size in 15 explanted hearts with ischemic cardiomyopathy. They found a good agreement between the two techniques (*r *= 0.89) although there was a slight overestimation with MPS. When comparing MPS and LGE in acute as well as chronic studies, Hedstrom et.al [19] found that the MPS perfusion defect was slightly larger than the hyperenhanced volume by LGE. Using a different tracer, ^201^Tl, a similar result was found by Lund et. al with MPS showing a slightly larger infarct extent compared to LGE (r = 0.73) in 60 patients with acute myocardial infarction [20]. Fieno et.al, comparing patients with chronic coronary artery disease, found a correlation of *r = *0.76 between rest ^201^Tl and LGE [21]. Interestingly, they found a higher correlation, *r *= 0.90, when comparing re-distribution ^201^Tl MPS and LGE in the same patient group. The authors suggested that the stronger relationship in redistribution ^201^Tl MPS is closely related to the potassium space which is assumed to be more reflective of the amount of viable myocardial cells. If so, tetrofosmin does not redistribute and cannot be expected to follow the potassium distribution. Our findings thus agree well with rest-^201^Tl MPS in the Fieno study [21].

The difference in infarct size between the MPS and LGE can be due to several physiological mechanisms being active in the different techniques. MPS represents the perfusion of the myocardium and the tracer uptake is dependent on the blood flow. In LGE, the myocardial scar is enhanced by the presence of extracellular Gd-DTPA and the concentration depends on the washout kinetics of the extracellular space [26,27]. Spatial resolution is approximately 10 times higher with LGE than with MPS causing a larger partial volume effect with MPS. This lowers the efficacy of MPS to detect subendocardial scar, but not for the detection of transmural infarcts [28]. Also various acquisition and reconstruction parameters such as choice of filters and filter parameters may affect the quantitative results in MPS [29]. Ibrahim et.al [6] found that the scar size with LGE depends on the inversion time. Maintaining a constant inversion time resulted in a decrease of both signal and infarct extent on late acquisitions. If the inversion time was adjusted, the image contrast stayed at a high level for 42 minutes (437% of remote area). In our study, all LGE recordings were performed within this time span.

In four of the six studies where MPS showed infarct size exceeding + 1 SD compared with LGE, MPS displayed hypoperfused myocardium in the inferior wall. Also, in three of the five examinations where MPS suggested myocardial infarction but LGE did not, there was reduced MPS perfusion in the inferior wall. Previous studies have shown that LGE has a higher sensitivity for a myocardial scar in the posterior – inferior region [30], especially in the setting of a nontransmural infarct [31]. McCrohon et. al found that only approximately 25% of patients with presumed inferior attenuation defect on MPS have abnormalities on LGE [32]. It is likely that diaphragmatic/soft tissue attenuation artefacts could be one explanation for the false positive MPS exams in our study. Another possible explanation could be a reduced wall thickness [33]. This was our visual impression in two of the six patients where the MPS infarct extent was > 1 SD larger than the LGE extent.

Left ventricular function can be expected to be linearly related to the volumetric infarct size [34]. If not, other mechanisms such as excessive reduction in left ventricular systolic function e.g. due to stunning or hibernation could be invoked. As expected, in this study left ventricular function, estimated from the calculation of wall motion score index from cine-MRI as well as from echo, correlated moderately with infarct size, assessed with both MPS and LGE.

### Limitations

The initial seven patients received "single dose" (0.1 mmol/kg bodyweight) of intravenous gadolinium, which is less than the standard dose but still provides excellent image contrast [35]. The results from this study show that the difference in infarct volume and extent between MPS and LGE in these seven patients was less than 1 SD. In some patients there was a considerable time span between the initial MPS and the LGE exams. However, there was no intercurrent cardiovascular event between the exams and the 5 patients with the longest time difference (> 64 days) did not show larger deviation in infarct size than those with a shorter time span. Patient selection was based on the presence of a perfusion defect, which in some patients could be due to an attenuation artefact, possibly weakening the correlation between infarct size with the two methods. However, after excluding patients where neither MPS nor LGE showed myocardial scar, the correlation between MPS and LGE changed very little, showing that the correlation was rather robust for the influence of attenuation.

The detection limit for LGE uptake is conventionally felt to be in the range of 1 ml, which could represent less than 1% of the myocardial volume. In MPS, the clinical cut-off for scar detection with PERFIT is set at 2% of the myocardial volume, which is considerably larger than with LGE.

Finally, the change in the acquisition protocol for MPS from 32 to 64 views with a reduction of acquisition time from 50 to 25s after the first nine patients was done in order to optimize MPS acquisition. This change foremost reduced streak artefacts and, of less importance, improved spatial resolution.

## Conclusion

MPS and LGE agree fairly well in the determination of infarct size in both absolute and relative terms, although infarct volume is slightly larger with MPS. This could be due to physical factors in the reconstruction algorithm such as filter choice and cut off, the absence of scatter correction, differences in the segmentation, or biological factors such as hypoperfused myocardium and wall thinning. The correlation between WMSI and infarct size was moderate which could be due biological factors as well as imperfection in the subjective assessment of wall motion.

## Competing interests

The authors declare that they have no competing interests.

## Authors' contributions

LR planned the study, participated in the investigations of a majority of patients, performed all measurements and the main part of writing the manuscript. PB participated in the planning of the study, performed investigations on some of the patients, performed measurements and reviewed the manuscript. JO was responsible for the scintigraphic evaluation and reviewed the manuscript. PGB was responsible for setting up the MR-method and for transferring images. He also reviewed the manuscript. BMA performed all MR investigations and reviewed the manuscript. SAS was responsible for the scintigraphic method and reviewed the manuscript. JE participated in the planning of the study, investigated some patients, performed all measurements and participated in writing the manuscript. All authors have read and approved the final manuscript.

## Pre-publication history

The pre-publication history for this paper can be accessed here:


